# Accessing healthcare during the COVID-19 pandemic: a qualitative exploration of the experiences of parents and carers of children with chronic illness to inform future policies in times of crisis

**DOI:** 10.1186/s12913-023-09452-1

**Published:** 2023-05-23

**Authors:** Dominic Reed, Ingrid Wolfe, Jenny Greenwood, Sapfo Lignou

**Affiliations:** 1 Institute of Women and Children’s Health, King’s College London, 1 Lambeth Palace Rd, South Bank, SE1 7EU London, UK; 2 Ethox Centre and Wellcome Centre for Ethics and Humanities, Nuffield Department of Population Health, University of Oxford, Big Data Institute, Old Road Campus, Oxford, OX3 7LF UK

**Keywords:** Chronic illness, Neurodivergence, Diabetes, Mental-health, Healthcare inequalities, Child-health, Covid-19, Child healthcare services, Paediatrics, Impact

## Abstract

**Background:**

The purpose of thispaper is to explore the experiences of parents and carers of children with chronic health conditions in accessing healthcare during the Covid-19 pandemic. Children with chronic conditions typically rely on both planned and unplanned care, and contact with healthcare professionals over extensive periods of time. Their distinct care needs render these children vulnerable to even to minor changes in healthcare provision. The wide-ranging care disruptions during the pandemic were therefore likely drastically to affect their health and wellbeing; an assessment of the effects of Covid-19 policies on healthcare access and quality of care delivered for this group is needed.

**Methods:**

From 25/01/2022 to 25/05/2022, four focus groups were held with parents/carers of children with diabetes, neurodivergence, mental health conditions, and medical complexities to explore their experiences in navigating the healthcare system during the pandemic. Interviews were transcribed and then subjected to thematic analysis using NVivo qualitative research software.

**Results:**

Our results indicate that children with chronic health conditions (and their parents/carers) experienced difficulties accessing healthcare during the pandemic. Problems with late diagnosis, prolonged waiting times, and deficiencies with telemedicine were identified, as were impacts of healthcare disruptions on children’s wellbeing, and the wellbeing of wider families. We found that children with neurodivergence and those with mental health conditions were particularly affected with their health needs repeatedly de-prioritised. Furthermore, the loss of contact with multi-specialty clinical teams profoundly affected parents and carers, leaving them feeling isolated in managing their children’s health. These diminished relationships became another vector for uncertainty in supporting children’s health.

**Conclusion:**

The effects of healthcare disruptions on the welfare of children with chronic conditions (and their families), are well evidenced in this work, providing deeper understandings of the relationships between these children, their families and clinicians. The evidence in this paper aims to inform future policy and ethical guidelines so that the needs of children with long-term health conditions can be properly considered in times of crisis.

**Supplementary Information:**

The online version contains supplementary material available at 10.1186/s12913-023-09452-1.

## Background

The United Kingdom experienced some of the highest COVID-19-linked mortality levels in Europe [1]. By June 2021, three national lockdowns had been instituted in efforts to contain transmission of the virus and reduce the overwhelming strain it was placing on the National Health Service (NHS).

Despite the large number of hospitalisations and deaths resulting from COVID-19 throughout all four UK nations, in general children and young people (CYP) aged 0–25 were considered less clinically vulnerable to the virus than adults, with much less critical illness resulting from COVID-19 among children [2]. However indirect impacts on CYP from the policy responses to the pandemic were notable.

During the first UK lockdown, Emergency Department (ED) attendance fell by 50% among CYP [3], and there was also a marked reduction in admissions to paediatric intensive care units (PICUs) in the UK and Republic of Ireland (ROI) [2]. These reductions in emergency attendance and admissions came without “measurable severe effects on child health” and have been ascribed to the effects of public health messaging (urging adults and children to “stay at home” and “protect the NHS”) and fears of contracting COVID-19 in healthcare settings [4].

The perception that children were less vulnerable to the pandemic meant that paediatric services (in terms of staff, as well as clinic and inpatient space) were regularly re-allocated to provide care for adult patients [5]. As a result, elective treatments, non-urgent care, and diagnostic services were suspended for the paediatric population. This rapid re-alignment of services led the Royal College of Paediatrics and Child Health (RCPCH) to argue that children “disproportionately suffered” [6] in terms of availability of care during the pandemic.

It is estimated that chronic conditions affect 13–27% of the paediatric population with consequences that endure into adulthood. Chronic health conditions are generally defined as those conditions that last more than 12 months and are severe enough to affect many aspects of life. They may create activity limitations, frequent pain and discomfort, hospitalisations, medical treatments, changing diet and lifestyle. Examples of chronic conditions include chronic illnesses (such as diabetes mellitus, asthma, congenital heart disease, attention-deficit hyperactivity disorder and depression) and chronic physical disabilities (such as visual or hearing impairments, cerebral palsy, loss of limb function).

Children with chronic conditions have continuous healthcare needs that include daily management and potential emergencies They rely heavily on coordinated planned care, supplemented by urgent care when needed. To help them manage their condition effectively and maintain their overall well-being, children with chronic conditions and their families regularly interact with a wide range of clinicians from different healthcare specialties, as well as professionals from other supportive services i.e. in social care and education. As a result of their specific needs and long-term reliance on complex services these children are particularly vulnerable [7] to changes in healthcare access. Available research suggests their care was significantly affected by the pandemic [8].

The longer term needs of children with chronic conditions were deprioritised as they were perceived to be less urgent due to their reduced vulnerability to the pandemic, leading several researchers [3] to argue for increased guidance for clinicians in providing paediatric care during the pandemic, stressing that such guidance was missing during the first waves of the virus.

This paper presents evidence that can inform guidance to support the needs of children with chronic conditions for future health systems shocks, and more broadly when decisions must be made about distribution of scarce resources in the face of competing priorities.

## Methods

### Design and setting

This study used a qualitative design, collecting data through focus groups that were then interpreted with thematic analysis. At the time the project was designed, there was a dearth of research exploring the healthcare experiences of children with chronic health conditions (and their families) during the COVID-19 pandemic in the UK. Therefore, there was no published data available on which to develop a hypothesis we could take forward to analyse through a large scale, quantitative assessment. Qualitative research (particularly using thematic analysis) has the benefit of not depending as heavily on extant hypotheses, but is instead a more inductive approach, allowing hypotheses to emerge from analysis of primary data [9]. As such, this was a suitable approach to assessing children’s and parents/carers’ experiences during the pandemic. The sensitivity of the topic under assessment, the fact that fieldwork had to be completed remotely and the (comparatively) small group of individuals applicable for joining the study further supported the value of a qualitative approach.

As the individuals responsible for managing the health of their children, parents and carers were best placed to recount families’ and children’s healthcare experiences during the pandemic. A purposive sample was chosen. Parents or carers had to meet the following eligibility criteria to participate in focus groups: (1) Be a parent or carer of at least one child with a chronic health condition aged 3 months to 17 years ; (2) have sought access to or used healthcare services for the health needs of a child with chronic condition before the pandemic (3) have sought access to or used child healthcare services during the pandemic; (4) be resident in the UK; (5) have sufficient English language skills; (6) consent to participate; (7) be happy to discuss their experiences/views regarding healthcare delivery for chronic illness during the Covid-19 pandemic. In order to capture diverse experieneces of accessing paediatric chronic care, before and during the pandemic, based on service, region or patients’ socioeconomic background, the research team aimed toinclude participants who were responsible for the care of children with varying health conditions and impairment levels, from diverse socioeconomic and educational backgrounds, residing in different regions of the UK. To facilitate recruitment and ensure a varied sample we liaised with a large UK-wide organisation supporting children and families who distributed recruitment materials through networks of professionals and patient organisations working with parents/carers newsletters and social media Later recruitment came from snowball sampling of existing participants to locate additional participants who may not have been reached through the initial recruitment efforts.

### Data collection

In total four groups were recruited: 1 group for parents of children with diabetes, 1 group for parents of children with complex conditions, 1 group for parents of neurodivergent children, and 1 group for parents of children with mental health conditions. These conditions were selected with different ‘axes of interest’ in mind. They captured (i) a range of ways in which children and families interact with the health and social care system, and (ii) children whose conditions represent different features (such as episodic vs. lifelong, static vs. progressive, stable vs. unstable) and level of complexity.

A total of 20 parents/carers consented to participate and took part, with up to 6 participants included in each group. Participants were mainly from England and Northern Ireland, with 1 from Scotland. Although a diverse sample was achieved, it was not necessarily representative of the wider population. This is consistent with the aims of qualitative research, which seeks to explore in-depth experiences rather than generalize to the entire population. The participants were parents and carers responsible for the care of one or more children with chronic health conditions, of both genders and varying ages (3–16 years old), with varying levels of health condition severity indicating a broad spectrum of health challenges (see Table 1). Participants from diverse educational and socioeconomic backgrounds were recruited, but the organization responsible for recruitment retained specific information on their socioeconomic status. To some extent, the participants’ socioeconomic background is reflected through their employment status, as shown in the table of demographics (Table 1). Only one parent or carer responsible for the care of a child with a chronic condition during the pandemic was asked to take part.

The interview topic guide was developed based on a literature review on the provision of and access to child health services during the pandemic, as well as consultations with key stakeholders, including child health professionals and children’s advocacy groups. Due to the sensitive nature of the research topic and the general difficulties associated with the pandemic, the research team decided to send a copy of the discussion guide to participants one week in advance to increase their sense of control and ensure their emotional wellfare during the discussion.Discussions were held remotely using videoconferencing software, from 25/01/2022 to 25/05/2022. and lasted for approximately 60 min. They were guided by a trained researcher with expertise in ethics and child health who used an open-ended interview guide (see Appendix 1). Questions addressed all participants and participants reflected on the questions and topics in turns. The discussions were audio-recorded and transcribed for analysis.

.

**Table 1:** Demographics

		Total
**Number of participants**		20
**Gender**		
Male		4 (25%)
Female		16 (75%)
**Occupation**		
Stay at home parent		3 (15%)
Full-time worker		9 (45%)
* Healthcare*		*3 (15%)*
* Education*		*2 (10%)*
* Charity and public service*		*3 (15%)*
* Other*		*1 (5%)*
Unknown		8 (40%)
**Type of carer**		
Parent		19 (95%)
Other family member		1 (5%)
**Country of residence**		
England		11 (55%)
Northern Ireland		7 (35%)
Scotland		1 (5%)
Unknown		1 (5%)
Wales		0
**Children’s age** (mean) (Range)		9.2 3–16
**Children’s health conditions**		
Type 1 diabetes		6 (30%)
Mental health condition (not specified)		3 (15%)
Autism Spectrum Disorder		3 (15%)
Autism Spectrum Disorder, Developmental Co-ordination Disorder and Diabetes Insipidus		2 (Q0%)
Autism Spectrum Disorder and Attention-Deficit/Hyperactivty Disorder		1 (5%)
Asperger’s Syndrome		1 (5%)
Anxiety		1 (5%)
Chronic liver condition		1 (5%)
Chronic medical complication secondary to Down’s Syndrome		1 (5%)
Congenital bowel disease		1 (5%)

### Data analysis

Focus group data was analysed using NVivo V12 Qualitative Analysis Software. Line-by-line analysis yielded initial codes which were then refined through consultation within the project team, with extraneous codes ultimately discarded.

In order to preserve the confidentiality of participants, identifiers were replaced on all project documents by an alphabetical code to which the study team retains the key.

### Ethics

This study was undertaken with the approval of the Medical Sciences Interdivisional Research Ethics Committee of the University of Oxford on the 24th of November 2021, (Ethics Approval Reference: R73852/RE001).

## Results

Focus groups with parents/carers of children with chronic illness and long-term conditions revealed nuanced descriptions of the relationships between parents/carers and clinicians in the management of children’s health, the importance of established therapeutic relationships and supportive mechanisms for children with chronic conditions and their families in times of crisis and how these were affected by the COVID-19 pandemic.

These findings emerged from focus groups with parents and carers who had a child or children with chronic illness, and who sought access to or used health services during the pandemic.

### Changes in healthcare provision

#### Remote consultations and insufficiencies

One of the main healthcare changes made to provision of care for patients during the COVID-19 pandemic was the use of phone and virtual (telemedical) consultations instead of in-person appointments. Many of the participants in this study reported experiences of virtual care. While a majority acknowledged the reasons for curtailing in-person consultations, they argued that telemedical consultations could not replicate face-to-face contact when it came to properly managing illness.

The move to online services with ‘unknown’ clinicians was regarded as challenging :

*“His [son’s] glucose levels were quite all over the place and we just needed someone to speak to. And, to be honest, considering the pandemic, we were struggling to get through to the team itself…. our appointments … they were all online, but there was always a different person that we were speaking to, which didn’t probably help”* (I, parent of a diabetic child).

The value of multi-specialty, in-person input was not restricted to the immediate medical interventions necessary to prevent serious illness. Instead, it appears that parents and carers valued the input from multiple healthcare professionals and saw them as an essential part of the care for their children, whether all the clinicians concerned were specifically ‘needed’ at each consultation. In the following excerpt a parent describes no longer having contact with the clinicians in the multi-specialty team treating their child as a ‘loss’ even while noting that the primary treating doctor, who they still saw during the pandemic, was effective:

“*We used to really look forward to them because it was meeting up with old friends almost. But that’s been taken away now and it’s just us and the consultant, who’s amazing, but yeah, the, the dynamic of our face-to-face appointments has now changed because you can’t get that many people in a room and social distance”* (Z, parent of a diabetic child).

The process of diagnosis was considered to have been particularly affected. Personal contact was essential to ‘unlock’ frank discussion about mental health:

*“…It was like, ‘Oh well, it’s been, it’s been five weeks now so we better give them a call…. And then there was not really any outcome. There was no immersive experience that you really need to have to open up especially as a child… I just found it was a barrier, especially when it’s mental health”* (X, parent of a child with mental health condition).

Phone consultations were considered responsible for inadequate referral to A&E:

“ .*If the GPs had just said, “Yes, come down and we will see them” or even, “Send us a picture or a video call” then we wouldn’t have had to go to hospital…. you hear more and more often with GPs the same thing, you know, [sending] people to the hospitals just by telephone consultation, you know, they’re not actually seeing the patients as much as they should”* (Y, parent of a child with bowel condition).

Several participants described telemedical appointments where children were not even present, sometimes because of children’s technology fatigue but also because appointment slots were difficult to obtain and so parents and carers had to ‘take what they could get’. This led many to argue that children were left out of consultations in a way that would not have occurred pre-pandemic.

“ *I did do some appointments without J and went through the numbers myself because, to be honest, he doesn’t really engage in the discussion over what we need to change and what we need to do to make his numbers better*” (O, parent of a diabetic child).

Despite the challenges of using remote consultation during the pandemic, parents and carers described some positive aspects of this approach, such as eliminating the need to take time off work or arrange for transportation to a healthcare facility. A few participants noted that remote consultations can make it easier for adolescents to attend medical appointments and receive the care they need:

*“He had about eight sessions of CBT and it seemed to work really well … when 11 you’re quite obsessed with computers it, it went down a treat, really”* (T, carer of a child with neurodivergence).

#### Access to information

A regular criticism among parents and carers in all focus groups was the reliability of COVID-19 information, both generally and in relation to their child’s specific condition. Participants described feeling ‘lost’, with regular clinical contacts disrupted and no clear idea of who to talk to about negotiating their child’s ongoing care., The following excerpt describes how this lack of certainty meant that parents/carers felt they had to determine safe COVID practices themselves:

*“The consultants, they didn’t have any information themselves, so we were all just winging it and just doing what we felt was safe for our own, for our own children”* (Z, parent of a diabetic child).

When, during the pandemic, parents and carers sought out reliable information from clinicians and this wasn’t available, it was deeply affecting. As in the following excerpt, most participants did not blame healthcare professionals for these issues:

*“I just think it was quite unsettling asking my son’s consultant, who’s a professor, like, “What is this [COVID-19] about? You know, just tell us anything.“ And he just would shrug his shoulders. He didn’t know anything…. You couldn’t find anything online that was from a reliable source. The JDRF [Juvenile Diabetes Research Foundation], the charities that normally will, you know, put a little bit of information, they had nothing on there [their websites] either”* (V, parent of a diabetic child).

V went on to describe the alternate sources of information she utilised in the absence of advice from clinicians. Notably, these alternate information sources included researchers in countries outside the UK:

*“I think there was one reliable source of information online from Dr Partha Kar. So I kind of trusted his tweets, so I would always keep alert and see what he published, research from China about Type 1, children with Type 1 and how they reacted with, to COVID”.*

For many, the dearth of information from clinicians (and other trusted healthcare sources like the JDRF) described by V profoundly impacted children and parent’s healthcare experiences during the pandemic, resulting in wrongful advice and even, in some cases, inappropriate care.

“My wee boy had a chest infection and we contacted the doctor, just basically to see any advice on his own, and we were told lateral flow him…. Then they said to go to a Covid testing centre on the other side of Belfast? …… When we got there we were literally ushered through Sellotaped floor paths into a room with a doctor who he didn’t know… looked at him for all of two or three minutes and then we were sent home again….. It made a really bad situation ten times worse” (K, parent of a neurodivergent child).

Finally, many participants raised concerns about the difficulties in accessing information related to vaccinations and in discerning the vulnerability categories that their children fit into. :

*“I’m not really clear about vaccinations and things for my son and his age group…. I haven’t heard anything about his age group or his condition. And I think somebody touched on earlier about, was he in a vulnerable category? Was he not?…. So a bit more information about that would have been helpful …. Maybe some sort of formal information from the hospital”* (M, parent of a diabetic child).

#### Delayed diagnosis

Participants across focus groups described the difficulty of children receiving a diagnosis during the pandemic, recounting their experiences, and referring to those of other families. Diabetic Ketoacidosis (DKA), a medical emergency, was identified as a particular problem, with several parents describing how the absence of clinician contact in this context affected parents and children.

*“ I think there was a worry about the children who were missed and diagnosed quite late on with things like diabetes. There was a lot more DKA occurring because people were frightened and mistaking the symptoms for maybe COVID, and there wasn’t a lot of evidence that children were actually getting COVID and being unwell at that point”* (O, parent of diabetic child).

As seen in the following excerpt, concerns regarding late diagnosis were not peculiar to parents of diabetic children. :

*“I’m not sure how much the liver team in [English Midlands] was affected, but definitely the teams in Belfast were…I know in terms of RCC [Renal Cell Carcinoma] and support again because [R’s son] was so acute there is a protocol that they have to follow in terms of his aftercare and checking his weight and people coming to see us and all that stuff. So we had that, but other people who, who didn’t have anything acute, it was, like, “Well, they’re not on that essential list anymore”* (R, parent of a child with complex liver condition).

There were further concerns too, from parents of children with complex conditions, that clinicians simply ‘took parents’ word’ for the state of children’s health, a position parents felt unqualified to occupy and which further blurred their role. This resulted in pre-existing problems worsening because of the pandemic.:

*“It ended up being about two years, and from that initial referral to being seen and diagnosed they’ve seen him once they whole time…. They relied on a telephone interview with me which, you know, they are just taking what you say for sort of gospel, they are not seeing your child*” (K, parent of a neurodivergent child).

#### Acute vs. planned care

While all participants described disruption to medical services, there was a widespread belief that acute care was better maintained during the pandemic, with treatment, clinician contact and reliable information essentially as accessible as before the pandemic. :

“*Because we were in a more acute setting then we had all of that [information] provided for us…. We were in hospital getting information, so [the] access you get because you’re there and sometimes they’re having to tell you and we had lots of face-to-face conversations*” (Q, parent of a child with medical complexities) .

Some participants noted that acute COVID-19 related services were particularly well maintained when compared with other aspects of the health system.:

*“Everything related to COVID was quite easy to access, so if you had a …… cough, loss of sense or taste or smell they were pretty quick off the bat, that was quite easy to get…. anything relating to COVID was quite quick. Getting seen by the GP, actually just getting into the GP was really difficult.”* (X, parent of a child with mental health condition).

In addition, while urgent care needed to prevent severe illness or death was maintained, even in an acute setting, the wraparound services that accompanied it were drastically curtailed. This was particularly evident when children required surgical interventions.:

*“The surgical staff [were] saying, “We just, we’re not doing standard procedures, we’re not doing OGD’s [Oesophago-Gastro Duodenoscopy], we’re not scoping, so if it’s not something critical and urgent we have no idea when we’re going to be allowed to add those children to our list”* (R, parent of a child with a complex liver condition).

R went on to describe the extent to which these wraparound services were affected, noting that standard check-ups following surgery were not upheld as regularly as required:

*“So children who should have been seen on an annual basis to check for the various things that needed watched in case they needed further care just were, were being left for months on end, so his [R’s son’s] follow-up care in [English Midlands****]****should have been a liver biopsy every year and he was 15 months post transplant before he had that ….”* (R, parent of a child with a complex liver condition)

The preservation of acute care over chronic care, while reported by many participants, was not evidenced across all focus groups. In the mental health group, participants were keen to stress that even urgent care was difficult to access. This challenge re-enforces evidence suggesting mental health services were particularly badly affected by the pandemic [10].

*“So we’ve got a crisis number to ring which I’ve used a couple of times… But when I ring it goes to a receptionist who says ‘The duty nurse isn’t at her desk right now, someone will call you back’……Which when your child’s in a crisis situation you haven’t really got the time to be hanging on for a phone call”* (X, parent of a child with mental health condition).

There were perceived differences between Northen Ireland and England as well, and Q spoke more generally about the availability of services for neurodivergent children, referring specifically to the perceived longer waiting times for children in Northern Ireland compared to England:

“I think it’s interesting that we’re all from Northern Ireland… I mean I saw something about the waiting lists in England, the NHS and I laughed. I know it sounds awful, but our waiting list for some specialties [in NI] are five years. Our waiting lists are so much longer than across on the mainland, so we already have massive backlogs.” (Q, parent of a child with medical complexities)

The service provision for all of the clinical groups included in the focus groups has not been ideal before the pandemic. Inequity in access to non-urgent care seemed rather to reflect pre-existing problems for children with neurodivergence and mental health challenges:

*“My son, he’s 12 and he’s got autism. He was diagnosed when he was three, and he only got seen twice and this was in 2013. So that doesn’t seem hugely different to in Covid, just still just sort of dumped and waved off, so.”* (T, parent of a child with neurodivergence).

### Impact on children and their families

#### Impact on children’s wellbeing

The discrepancy between acute and chronic services led several participants to argue that children’s health had to reach a ‘crisis point’ before they could access care, something that occasionally meant children and parents were put through unnecessary distress. Again, the level of disruption was manifestly higher for children with complex conditions. :

*“It was so frustrating… having these awful, awful, awful experiences at home where everyone by the end was suffering. Just seeing him suffer so badly for him to go to the place that we were supposed to be getting the help and support from and her [the therapist] giving me the feedback, ‘Oh, well he’s fine.’….And we’re like, ‘Well that’s not consistent with what we’re seeing at home.’ And that just wasn’t taken into account until we got to crisis point”* (X, parent of a child with mental health condition).

In addition to issues arising during the pandemic, many participants discussed the downstream effects of restricted healthcare access on children’s future life chances. These reflections took several forms but chiefly concerned the extent to which children’s lifestyles had been affected by the pandemic and the disruption to care previously shared between health and other community services, potentially leading to increased health deficits in the future.

In the following excerpt, K suggests that delays in children receiving appropriate care, and the subsequent impact on wellbeing would eventually burden adult health services:

*“I mean, if the work is done early enough you can avoid them [children] having to need the other services down the line, but the bottleneck is so huge that all we’re doing is storing up problems for adult services”* (K, parent of a neurodivergent child).

#### Parent’s new responsibilities

While stressing the effect of the pandemic on children’s wellbeing, participants also described how COVID-19 disruptions affected family life and their responsibilities. Across participant groups, parents felt their roles changed during the pandemic, with additional burdens placed upon them to monitor and support children’s health. In some cases, these responsibilities extended to assisting in the examination of children in consultation with clinicians. Perhaps unsurprisingly, few parents were comfortable with this, fearing the potential impact on children’s health. :

*“We had one experience with the GP with one of my other children and I’m not medically trained and I rang her with concerns, she was asking me these questions over the phone, asking me to, you know, feel here and touch here and all that and we were sent to hospital with suspected appendicitis, we sat in hospital for a couple of hours… and he was diagnosed with tonsillitis not appendicitis”* (Y, parent of a child with bowel condition).

More frequently, parents and carers’ negative experiences related to increased responsibility for navigating the complexities of the health system, after losing contact with clinicians who supported them with this pre-pandemic. This was particularly trying for parents of children requiring multi-specialty input. Q discussed how difficult it was to manage specialty waiting lists:

*“I got three different letters from three different specialties saying, “Does your child still need to be on this waiting list? Please phone us on the following number or please go to this website and fill out a questionnaire, if you don’t do it by Friday your child will be removed from the list.”* (Q, parent of a child with medical complexities).

In these reflections, it became clear that a lack of clinician contact profoundly impacted parents/carers’ confidence in managing their children’s health.

*“You actually have to turn into somebody that you don’t even recognise ‘cause you are their biggest pain in the arse that anyone has ever come across because you have to just keep on going and keep on going, and keep reiterating the same thing over and over again”* (P, parent of a neurodivergent child) .

While many participants described taking on further duties relating to children’s health, a majority also noted that they felt ‘unqualified’ for these tasks, resulting in feelings of ‘guilt’. They reflected on their own parenting abilities and spoke about their need to work to ensure that their child was ‘spoken for’ in the healthcare system, giving some indication of the increased role parents/carers had in negotiating the health service during the pandemic.

## Discussion

Despite the general commonality of experiences across focus groups, there were indications that children’s pandemic healthcare experiences differed in some ways depending on the chronic condition they had. The most notable findings relate to the experiences of neurodivergent children and children with mental health conditions. Children in these groups experienced the greatest level of healthcare disruption during the pandemic, an important conclusion given that services for these children were already under considerable strain before the pandemic began. A second key finding was that children with long-term health conditions generally encountered heavily disrupted care except in acute medical emergencies, when healthcare was mostly (although not always) less disrupted.

Initially, disruptions appeared to result in late diagnoses, with individuals in the neurodivergent and mental health groups discussing this in detail, however, as noted above, these concerns were also raised by parents and carers of diabetic children, frequently focused on the specific issue of DKA.

The starkest difference in experiences between parents and carers of children with different long-term or chronic health conditions came in the level of disruption and even alienation felt by the parents of neurodivergent children and children with mental health conditions compared with parents/carers in the other focus groups. While participants in all groups noted that COVID-19 greatly affected their interactions with the health service, it was parents and carers in those two groups that reported the most profound changes. It was also those particiapants that, in general, experienced the longest delays in accessing clinicians and obtaining diagnoses, just as it was those participants that were most frequently faced with efforts to discharge their children from specialty waiting lists; in their opinion almost always prematurely.

For parents and carers of neurodivergent children and children with mental health conditions, there was also the widely held belief that services were affected because of the nature of their children’s conditions. During discussion, it became clear that they considered that their children’s health was specifically de-prioritised during the pandemic, the result of a stigmatising system that refused to consider mental health and neurodivergence as analogous to ‘physical health’. These participants asserted that even before the pandemic, services for mental health and neurodivergence were not adequately funded and indeed were the most likely groups to stress that services were inadequate before the pandemic hit. This dissatisfaction was typified by individuals in the mental health group challenging the notion that acute care had been well maintained during the pandemic, in stark contrast to participants across all other groups. These conclusions find strong support in the available literature, including the recent Lancet Commission on stigma and discrimination in mental health [11], which asserted that patients with mental health conditions routinely receive poorer healthcare than those with physical conditions.

Accordingly, it appeared that parents and carers in the neurodivergent and mental health focus groups felt a greater obligation to ‘do more’ personally to ensure their children had access to appropriate care. Given the frequent difficulties experienced by parents and carers who took on these roles, it was therefore also more likely for parents and carers of children with neurodivergent or mental health conditions to report greater frustration with healthcare services during the pandemic.

Some differences between participant experiences were also observed depending on their geographic location. These differences were limited but mostly experienced by participants from Northern Ireland (NI), specifically parents of neurodivergent children. The small sample size of the study limits our ability to draw definitive conclusions about the health system in NI. It is possible that the reported issues reflect the opinions of participants rather than actual systemic issues, or that they are due to the smaller scale of health services in NI compared to more populous parts of the UK, which may be more similar to remote or rural services in other nations Neither conclusion could be reliably determined from the data, but the responses of participants offer opportunities for future study. Participants in all groups and from differentregions of the UK (Northern Ireland, England and Scottland) experienced delays in diagnosis, treatment, and general clinical interactions because of the COVID-19 pandemic. However, some participants from NI argued that delays were often longer there, and, as a result, parents and carers were more likely to seek advice/treatment from clinicians in other UK nations. This did not always result in individuals travelling outside of NI for care, but did lead to some situations where, in particular, English services were considered by parents and carers. That sentiment was expressed well in an exchange between two participants relating the difficulties in accessing various services for neurodivergent children in NI, that ended with one parent suggesting that another try and access a charity in England for assistance. While these participants merely discussed accessing care elsewhere, another parent (in the complex conditions group) did actually need to travel outside of NI in order for their child to receive treatment in the English Midlands, after which the child spent a prolonged period of time in hospital there.

As noted, parents of children with different long-term health conditions did, on occasion, report varying healthcare experiences during the COVID-19 pandemic^1^. In the main however, there was a discernible homogeneity to responses in most contexts, regardless of children’s health conditions, with parents across all groups reporting similar concerns. This suggests that children with chronic illness (and their families) share common perspectives in terms of their relationships with the health service and individual clinicians, and that these relationships, though affected to different extents by the pandemic, led to similar perceptions of disruption.

It is evident from the data that acute care was better preserved during the pandemic than chronic care requiring multi-specialty input (or at least this was perceived to be the case). Importantly, these conclusions were reached not only from parents of children requiring routine care, but also included parents whose children needed acute care during the pandemic. These understandings were sufficiently nuanced that parents even pointed to areas in which acute and chronic care interacted, i.e. in aftercare following surgery, as evidence of a space where disruption and deficiency became more pronounced.

As a result, it appears that there was a recognised effort by clinicians and the health system generally to safeguard acute care by rerouting staff and resources from planned and non-urgent care, something recognised by parents and children interacting with services. The experiences reported here support the arguments advanced elsewhere that acute care in the UK was mostly preserved during the pandemic and fits in with the general argument that the health service is a reactive rather than a proactive entity [12]. This preservation of the essential was not limited to acute services, but was described within the context of chronic care where interactions with clinicians were reduced from multi-disciplinary meetings (e.g. with nurses, dietitians ) to consultations with individual doctors. Given doctors’ roles as directors of diagnosis and treatment, parent’s/children’s contact with them was maintained (even if only through online/telephone consultation), when contact with other clinicians lapsed.

There was widespread reflection among participants in all groups about how parent’s roles had changed during the pandemic, and how these changes had affected their perceptions of their ‘success’ as parents. Nearly all participants, regardless of their child’s condition, described the loss of one-to-one interactions with clinicians as one of the most disruptive aspects of the pandemic on their health service experiences. In some ways parents described this ‘ loss’ in terms of the practical issues it caused. Many individuals referred to problems with obtaining diagnosis, with empathy expressed in particular for those families experiencing a new diagnosis during the pandemic, when lack of in-person consultation could greatly affect illness management. Still others cited the difficulties experienced in navigating a complex health system without the assistance of clinicians they had grown to trust.

But for others, the lack of one-to-one contact with clinicians was more profound. It became clear that for many parents of children with long-term or chronic conditions, clinicians were a vital source of help and support that they received nowhere else. Oftentimes, the nature of children’s conditions meant that pre-pandemic, they had regular interactions with clinicians, building deep and lasting relationships. For many, it was clear that they saw these relationships not only as a means to obtain treatment but also as barometers of their own parenting abilities when it came to supporting their child’s health. The prospect of health services shifting to virtual appointments as normal post pandemic care therefore has stark implications.

With the significance of these relationships outlined, it is perhaps unsurprising that profound changes were perceived so negatively. When parents and carers were asked in some cases to ‘act’ like clinicians in describing children’s symptoms, or even to undertake a role in examination and diagnosis, there was a deep and unwanted shift in the clinical relationship that led to self-doubt and self-criticism of parent/carer’s ability to support their children. In this context, instead of providing support, clinical relationships had mutated into another vector for uncertainty in tackling often difficult, long-term illness.

One uniting theme was that, in the absence of what might be termed ‘standard clinical contact’ parents and carers commonly referred to online forums and support groups to provide ‘reliable’ COVID-19 information. This recourse to alternate sources was observed across all focus groups, with participants noting the particular value of sites/forums run or inputted by other parents/carers. This ‘peer’ information network was relied upon by many, and while it undoubtedly had drawbacks (the risks of contrary/misinformation being paramount) it was heavily used as a result of pandemic disruptions. The value of other parents/carers’ information/experience likely extended from the ‘expert patient’ role that many participants occupied themselves when it came to their children’s health.

For children with chronic health conditions, and their carers, contact with healthcare and other supportive services is a regular occurrence, and participants had extensive experience (often built up over years) of interacting with a wide range of clinicians across different medical specialties. They accordingly had awareness of pre-existing issues in care provision for children in the UK and hoped that exposure of these issues during the pandemic led to much needed improvement.

Before the pandemic, service provision for all clinical groups included in the focus groups, drawn mainly from Engalnd and Nothern Ireland, was not ideal. However, the COVID-19 pandemic and the inadequate response to it have provided an opportunity to reassess how services are provided, especially for clinical and population groups that systematically face inequities in healthcare access and experience. To address these issues, healthcare providers could develop effective hybrid services that utilise digital health and telemedicine technologies to overcome geographic barriers while maintaining the benefits of in-person care.

Rather than returning to previous models, a hybrid model may offer numerous benefits for children with chronic health conditions and their families, such as enhancing monitoring and management and helping healthcare professionals to identify potential issues early on and provide timely interventions. Such a model may better engage parents/carers and older children in their own healthcare by providing them with access to educational resources, tools for self-monitoring, and platforms for communicating with healthcare professionals. This could help establish Shared Decision Making (SDM) as a routine process of care, in turn helping support decisions about healthcare options that best align with the patient’s values, preferences, and goals.

To ensure successful and fair implementation of hybrid models, healthcare providers should conduct a thorough assessment of patient needs to determine which services are most appropriate for remote care and which services are best delivered in person. This is particularly important as the potential cost-savings of remote care is likely to be attractive to policy-makers. Parents and carers should be actively involved in the design and implementation of the hybrid service to ensure that it meets the needs of both the child and their family. Clear protocols should be developed for patients, healthcare providers, and administrative staff to ensure seamless communication and coordination between in-person and remote care. This should include guidelines for how and when to use digital health and telemedicine tools as well as staff training in technical skills, patient communication and engagement. Ongoing support should be provided to patients and families to ensure that they are comfortable and able to use digital health and telemedicine tools.The specific issues in healthcare access that have been identified in this study have supported conclusions drawn from existing international research. Specifically studies have highlighted the disproportionate impact of the COVID-19 pandemic and the resulting measures on vulnerable populations, including children with special healthcare needs, and those with mental health conditions [13]. Additionally, children with chronic conditions have reported higher levels of anxiety, depression, and stress during the pandemic, compared to healthy children [14]. The pandemic has also had a significant impact on the mental health of both parents and children with medical complexities in Canada who experienced disruptions to healthcare services, including cancelled or delayed appointments, reduced access to specialist care, and difficulty accessing medications and medical supplies [15].

Other findings however, have been more novel. The effects of healthcare disruptions on parent (and wider family) wellbeing, are well evidenced in this research, providing deeper understanding of the relationships between children with chronic health conditions, their parents, and clinicians. Finally this work has added important detail to how and why delayed diagnosis of serious chronic health conditions has been such a significant feature of pandemic disruptions, identifying DKA as a particular cause for concern, something observed in the quantitative literature [16, 17].

## Conclusion

The need for clear and consistent guidance on how health systems should respond in their efforts to maintain public health and protect the most vulnerable members of society, including children with chronic and complex conditions, became obvious in this pandemic. By employing a qualitative approach this research gathered in-depth lived experiences of the carers of children with a range of chronic conditions. Accordingly, the conclusions drawn from this work can inform further research on how to better support and respond to the needs of these children and their families in times of crisis. In the short-term, this work will form part of a wider project aimed at assessing the ethical implications of implemented public health restrictions during the COVID-19 pandemic on vulnerable populations.

## Electronic supplementary material

Below is the link to the electronic supplementary material.

Supplementary Material 1

## Data Availability

The datasets used and/or analysed during the current study are available from the corresponding author on reasonable request.

## Footnotes

1 Although this study did not specifically investigate regional differences, we acknowledge the variations in healthcare systems across the four nations of the United Kingdom. Each nation has its own National Health Service (NHS) that operates under different administrative structures and policies, which can affect the provision of care for individuals with chronic conditions. Additionally, it is worth noting that the management of chronic conditions and the response to the COVID-19 pandemic varied across the four nations, which could have impacted the experiences of the participants in this study.

## List of Abbreviations

NHS: National Health Service
CYP: Children and Young People
OGD: Oesophago-Gastro Duodenoscopy
NI: Northern Ireland
DKA: Diabetic ketoacidosis
