# Supplementary material for: Accessing healthcare during the COVID-19 pandemic: a qualitative exploration of the experiences of parents and carers of children with chronic illness to inform future policies in times of crisis
Source: BMC Health Serv Res. 2023 May 23;23:530. doi: 10.1186/s12913-023-09452-1 (PMC10205033; doi:10.1186/s12913-023-09452-1)
Supplement: Supplementary file 1 — Supplementary Material 1 [file 12913_2023_9452_MOESM1_ESM.docx]

**Topic guide – Healthcare for child chronic illness during the Covid-19 pandemic**

We are interested to hear from you about your experience of getting healthcare for your child during the pandemic. We are especially interested in how you have felt about your child’s health and healthcare during this time and how well you feel the health system has been able to look after them.

Please feel free to talk about any experiences you have had since the first lockdown in March 2020 up until today.

Routine care/use of emergency services during the pandemic

1. Firstly, we’d like to know if anyone has seen their child’s GP or specialist team during the pandemic. Could you tell us why you needed to see the GP or specialist team and how did the appointment go?
2. Did you need to go to A&E for your child during the first lockdown or since then?

Changes in routine care delivery

We are interested to know if you child’s healthcare changed in any ways since the pandemic started.

Has the person delivering your child’s care changed more than usual? How do you feel about this?

1. Since the first lockdown in March 2020, have doctors or other medical professionals seen your child less frequently than before the pandemic started?
2. Has the frequency or duration of the your child’s healthcare appointments changed? Have the appointments been more or less frequent? Longer or shorter?
3. Do you think anything was missed in terms of your child’s healthcare because they weren’t able to see their medical team as often usual?

Perceived barriers and facilitators to care seeking

This next section is about how you got hold of information about healthcare during the pandemic.

1. Which have been your main sources of information regarding COVID-19?
2. Did you feel you had enough information and if not what kind of information were you/have you been missing? Can you think of anything in particular that would have been helpful to know?

Perceived barriers and facilitators to access to healthcare

This next section is about how easy or difficult it was to access the healthcare your child has needed during the pandemic.

1. Could you tell us about your experience trying to access healthcare for your child since the pandemic started?
2. If you have experience of using more than one of these services, were some more difficult to access than others? If so, what were the main differences and which healthcare services did you find more difficult to access?
3. Which healthcare services did you find easier to access?
4. If some of the services were easier to access, what made them easier?

Telemedicine

1. Did you have experiences of online appointments with the GP eg. on the telephone or video consultation?
2. Did you feel these video or telephone conversations were helpful for you and your child?
3. Did anyone have any particular challenges when trying to get support for their child through video or telephone consultations?
4. How would you feel about having video or telephone consultations from now on instead of face-to-face?

Ending questions

1. Of all the things we discussed, what to you is the most important? Is there anything that we haven’t discussed that you think is important and would like to add?
2. Have we missed anything?
